# Multi-Level Evaluation of Antioxidant Activity, Aging-Related Physiological Functions, and Longevity Effects of *Nelumbo nucifera* Gaertn. Embryo Extract, *Pleuropterus multiflorus* (Thunb.) Turcz. ex Nakai Stilbene Glycoside, and Pterostilbene

**DOI:** 10.3390/molecules31172981

**Published:** 2026-08-26

**Authors:** Kuo-Ching Jan, Jung-Chun Tai, Xiao-Zhong Liu, Yu-Mei Li, Mohsen Gavahian

**Affiliations:** 1 Department of Food Science, National Pingtung University of Science and Technology, Neipu, Pingtung 91201, Taiwan; 2 Chiayi Industry Innovation and Research Center, Chiayi 60002, Taiwan

**Keywords:** bioactive compounds, functional foods, extraction, phytochemicals, lifespan extension

## Abstract

Despite known antioxidant activity, the effects of plant-derived compounds on aging-related physiological functions and mechanisms remain unclear, which necessitates investigation at cellular and organismal levels. To examine whether these compounds can mitigate age-related physiological decline, *Nelumbo nucifera* Gaertn. (sacred lotus) embryo extract–water-soluble fraction (NEW), stilbene glycoside from *Pleuropterus multiflorus* (Thunb.) Turcz. ex Nakai (PM-SG), and pterostilbene were investigated through chemical profiling, antioxidant activity, HepG2 liver cells, and *Caenorhabditis elegans* longevity assays, while principal component analysis (PCA) elaborated on the results. Quantitative LC-MS revealed that NEW extract was rich in alkaloids (neferine and liensinine), while PM-SG mainly contained 2,3,5,4′-tetrahydroxystilbene-2-*O*-β-D-glucoside. PM-SG showed the strongest radical-scavenging activity (DPPH, TEAC, and ORAC), followed by pterostilbene. However, pterostilbene exhibited the strongest cellular antioxidant activity among the three test materials (NEW, PM-SG, and pterostilbene), an effect attributed to its superior cellular uptake, although it was less potent than the reference standards EGCG and quercetin. All samples extended the lifespan of *C. elegans* dose-dependently, with the strongest effect from pterostilbene (40–50%), then PM-SG (25–35%) and NEW (18–22%). Pterostilbene and PM-SG improved health measures, including enhanced resistance to heat and oxidative stress, and maintained feeding function. Discrepancies between in vitro antioxidant rankings and observed cellular effects indicate that radical-scavenging activity alone cannot fully predict in vivo anti-aging efficacy, which depends on cellular uptake, activation of endogenous defense mechanisms, and modulation of longevity pathways. Further clinical studies are needed to determine the potential relevance of these findings to human health.

## 1. Introduction

Aging is characterized by the progressive accumulation of cellular and molecular damage, culminating in functional deterioration and increased susceptibility to disease. The free radical theory of aging posits that this process is predominantly driven by the accumulation of reactive oxygen species (ROS). However, it is important to recognize that ROS also serve critical roles as signaling molecules, and their complete elimination may disrupt normal cellular functions. Consequently, maintaining redox homeostasis, rather than maximizing ROS scavenging, may represent a more efficacious strategy for decelerating the aging process [1]. In aging organisms, ROS are generated from multiple sources, primarily the mitochondrial electron transport chain, with additional contributions from NADPH oxidases and peroxisomal oxidases. As antioxidant defenses decline with age (particularly in post-mitotic tissues), the oxidative damage to lipids, proteins, and DNA escalates, thereby accelerating cellular senescence.

NAD^+^-dependent sirtuins (SIRT1, SIRT3–5) play a pivotal role in modulating aging by regulating metabolic processes, mitochondrial function, and genomic stability. A reduction in NAD+ levels can initiate a feedback loop that exacerbates senescence. Empirical evidence indicates that overexpression of SIRT1 extends lifespan across various species, and compounds such as resveratrol activate SIRT1 via NAD^+^-dependent pathways [2]. Mitochondrial dysfunction, characterized by mitochondrial DNA (mtDNA) damage and impaired autophagy, is also a critical factor in aging. The AMPK/mTOR signaling axis governs mitochondrial quality control, with AMPK promoting mitophagy and mTOR inhibition enhancing autophagy. SIRT3–5 epigenetically regulate respiratory enzymes, and targeting these sirtuins may improve mitochondrial function [3]. Furthermore, several anti-aging compounds have been isolated from traditional medicinal plants.

*Plumula Nelumbinis* contains alkaloids, flavonoids, and polysaccharides that contribute to its antioxidant capacity [4]. Its water-soluble fraction has demonstrated free radical scavenging activity and lifespan extension in model organisms [5], although the precise bioactive constituents remain to be fully elucidated. Stilbene glycosides derived from *Polygonum multiflorum* exhibit potent antioxidant effects and may activate the SIRT1/FOXO1 signaling pathway, thereby promoting autophagy and attenuating neuroinflammation [6]. These compounds also induce ferroptosis in senescent cells, suggesting their potential utility as senolytic agents. Pterostilbene, a resveratrol analog with substantially higher bioavailability [7], can be further optimized through nanoparticle-based delivery systems [8]. It activates AMPK, stimulates autophagy, and extends lifespan in Drosophila and *C. elegans*, particularly when administered early in life [9,10,11]. Notably, polyphenols exhibit dose-dependent effects: low concentrations tend to activate protective pathways such as SIRT1/FOXO1, whereas higher doses may induce ferroptosis in senescent cells [12]. Whole extracts rich in polyphenols often elicit broader biological effects than isolated compounds, probably due to multi-target interactions [13], and they can restore autophagic flux, thereby reducing the secretion of senescence-associated factors [14].

Despite these advancements, several challenges persist in the field. These include a paucity of direct comparative studies, limited translation of in vitro antioxidant findings into demonstrable lifespan extension, and an incomplete understanding of structure-activity relationships and the optimal timing for intervention. Therefore, the present study was planned around two complementary research gaps that need to be addressed. The primary item is a structurally homogeneous QSAR investigation restricted to the stilbenoid compound class—TSG and oligomeric stilbenes (Multiflorumiside H) from *Pleuropterus multiflorus* (Thunb.) Turcz. ex Nakai (PM-SG), pterostilbene, and resveratrol as a structural reference, which ensures mechanistic coherence across the QSAR dataset. The secondary item is a multi-level biological comparison in which the NEW fraction (the aqueous extract of *Plumula Nelumbinis*, a pharmacopeial-recognized alkaloid- and flavonol-rich material with a documented safety record in the Chinese Pharmacopoeia) is included as a structurally contrasting reference class to test whether longevity-promoting effects are exclusive to stilbenoids or also extend to preparations with a different phytochemical profile. Preliminary *C. elegans* screening data showing statistically significant lifespan extension from the NEW fraction provided direct experimental justification for its inclusion. Both sources are long-established pharmacopoeial materials; their selection was guided by established traditional use rather than the novelty of the botanical source. Together, addressing these points can contribute to clarifying the relationship between chemical composition and multi-level biological activity, thereby informing the rational selection of natural product candidates for healthy aging.

Accordingly, the aims of this study include (1) to evaluate the in vitro antioxidant capacity of the test materials using multiple complementary assays; (2) to quantify their principal bioactive constituents by LC-MS; (3) to compare their protective effects against oxidative stress in cellular models; (4) to assess their impact on lifespan and stress resistance in *C. elegans*; and (5) to develop and validate QSAR models for the stilbenoid series that mechanistically link molecular descriptors to observed biological activities.

## 2. Results and Discussions

### 2.1. LC-MS Analysis and Phenolic Compounds

LC-MS analysis revealed distinct chemical profiles for the NEW and PM-SG extracts, consistent with their divergent biological activities. Quantification of principal bioactive constituents was performed using a high-resolution Orbitrap mass spectrometer employing selected reaction monitoring to ensure precise measurement. The chemical structures of the major identified compounds are shown in Figure S2 (see Supplementary Materials).

Supplementary Figure S1 presents chromatographic data. The TIC, scanning the mass-to-charge ratio (*m*/*z*) range of 100–1000 over a 50 min runtime, exhibited numerous sharp peaks indicative of a complex chemical mixture (Panel A). Selected reaction monitoring chromatograms (Panels B–E) monitored specific ion transitions (835→741, 851→741) to verify the presence and quantify various multiflorumiside isomers. Detailed fragmentation patterns, summarized in Table S2, provided critical structural insights. The *m*/*z* 741 fragment functioned as a diagnostic ion for the H, I, and J series, corresponding to a monosaccharide moiety following dehydration (C_6_H_10_O_5_ − H_2_O = C_6_H_8_O_4_), facilitating elucidation of glycosidic linkage sites. The *m*/*z* 611 fragment served as a secondary marker indicative of diglycoside cleavage. Additional ions, such as *m*/*z* 1481 (attributed to a sodium adduct cluster) and *m*/*z* 1221 (a rearrangement product), further clarified fragmentation pathways. These data underscore the structural diversity and isomer-specific profiles of multiflorumisides, which correlate directly with the distinct bioactivities observed in the NEW and PM-SG extracts [15].

The NEW extract was characterized by a high concentration of alkaloids, predominantly bisbenzylisoquinoline derivatives. As detailed in Table S2, the extract contained neferine (2874 ± 124.1 µg/g), liensinine (2140 ± 85.14 µg/g), and isoliensinine (1487 ± 57.42 µg/g), cumulatively exceeding 6500 µg/g. Additionally, significant levels of flavonoids were detected, including kaempferol (2437 ± 78.67 µg/g), quercetin (1420 ± 75.14 µg/g), and isorhamnetin (1230 ± 64.42 µg/g). This phytochemical profile aligns with established literature on *Nelumbo nucifera* Gaertn. The principal alkaloids—neferine, liensinine, and isoliensinine—are recognized for their neuroprotective properties, mediated through antioxidant mechanisms, inhibition of chronic inflammatory pathways (notably the NF-κB signaling cascade), and modulation of neurotransmitter systems [16]. The core structures of neferine (a bisbenzylisoquinoline alkaloid) and the flavonoids are presented in Figure S2.

The PM-SG extract exhibited a simpler chemical composition dominated by stilbene compounds. Table S2 indicates that 2,3,5,4′-tetrahydroxystilbene-2-*O*-β-D-glucoside (TSG) was present at a markedly high concentration (14,270 ± 175.1 µg/g), constituting approximately 95% of the total stilbene content. Notably, bisbenzylisoquinoline alkaloids were absent from this extract. In addition to TSG, a series of multiflorumisides were detected at lower concentrations, ranging from multiflorumiside H (275.8 ± 11.45 µg/g) to multiflorumiside L (20.64 ± 1.247 µg/g). Although these compounds are less extensively characterized than TSG, their potential bioactivity warrants further investigation. The chemical structures of TSG (a stilbene glucoside) and pterostilbene (a dimethoxylated stilbene) are also provided in Figure S2 for structural comparison.

### 2.2. Chemical Antioxidant Capacity

Four complementary assays—DPPH radical scavenging, TEAC, ORAC, and reducing power—were employed to evaluate the chemical antioxidant capacity of NEW, PM-SG, and pterostilbene (Table 1). Each assay captures a mechanistically distinct dimension of antioxidant activity: the DPPH and TEAC assays primarily reflect hydrogen atom transfer (HAT) and single-electron transfer (SET) capacity, respectively; the ORAC assay quantifies peroxyl radical chain-breaking ability under lipid-phase-relevant conditions; and the reducing power assay measures electron donation to ferric iron [17]. A notable observation across assays is the ranking inversion between DPPH and ORAC for TSG and pterostilbene—a pattern consistent with mechanistic expectations rather than a measurement inconsistency and one that is interpretable within the QSAR framework developed in Section 2.5. In the DPPH assay, PM-SG exhibited the lowest IC_50_ (7.57 ± 0.12 µg/mL), a result attributable principally to the structural properties of its major constituent, TSG. TSG possesses two ortho-dihydroxyl (catechol) groups on its stilbene aglycone—a structural feature identified in the QSAR model for chemical antioxidant capacity (Equation (3), Section 2.5.4) as the strongest single contributor to DPPH activity (standardized β ≈ 0.72) [18,19]. The catechol arrangement is established to facilitate proton-coupled electron transfer and to yield resonance-stabilized semiquinone radical intermediates, thereby enhancing the thermodynamic stability of the oxidized product [17]. Glycosylation at C-2 of TSG is thought to introduce only modest steric perturbation to this radical-stabilizing pathway [20], an effect reflected in the OHPS descriptor in Equation (3) (β = 0.18). In contrast, pterostilbene lacks catechol groups due to C3/C5 dimethoxy substitution, yielding a slightly higher DPPH IC_50_ (9.85 ± 0.26 µg/mL), consistent with the QSAR prediction [18]. The bisbenzylisoquinoline alkaloids in NEW show the weakest DPPH activity (IC_50_ = 23.08 ± 1.48 µg/mL), probably because the nitrogen atoms in these alkaloids may reduce electron density at phenolic hydroxyl positions, limiting SET efficiency [17]. In the TEAC assay, (+)-catechin ranked highest (11.28 ± 0.16 mM TE), attributable to its catechol A-ring architecture and low oxidation potential, which favor SET [21].

PM-SG (5.90 ± 0.97 mM TE) and pterostilbene (5.09 ± 0.86 mM TE) were comparable, while NEW scored substantially lower (2.21 ± 0.47 mM TE). The ORAC assay produced a ranking inversion: pterostilbene (16.46 ± 0.42 mM TE) exceeded PM-SG (12.47 ± 0.12 mM TE) despite PM-SG’s superior DPPH activity. This reversal may be partly attributable to the lipophilic C3/C5 dimethoxy substituents of pterostilbene, which could promote its partitioning into the hydrophobic phase of the fluorescein-based reaction system, thereby enhancing peroxyl radical interception at the aqueous–lipid interface [22]—an advantage less accessible to the more hydrophilic TSG (logP = 1.23 vs. 3.48 for pterostilbene), whose glucosyl moiety is expected to favor the aqueous phase [20]. This observation is consistent with the positive contribution of the conjugated π-system length descriptor in Equation (3) (β = 0.41), though the relative contributions of lipophilicity and π-conjugation to the ORAC result cannot be fully disentangled from the present data. The reducing power assay followed a comparable order—(+)-catechin > pterostilbene > PM-SG > NEW—consistent with the established importance of catechol groups and extended π-conjugation for electron donation capacity [19,21].

According to the above-mentioned observations, the cross-assay ranking patterns are interpretable within the QSAR framework. The C3/C5 dimethoxy substitution that reduces pterostilbene’s direct radical-scavenging performance relative to TSG also lowers its TPSA to 41.5 Å^2^, a key physicochemical property reflected in the negative TPSA coefficient of the CAA QSAR model (Equation (1), β = −0.024) and associated with superior membrane permeability and cellular antioxidant activity. The apparent trade-off between HAT efficiency and membrane-mediated peroxyl radical scavenging is structurally interpretable and consistent with established stilbene SAR principles [23]. The antioxidant assay data in this study are not merely descriptive but are mechanistically informative and quantitatively consistent with the QSAR models developed in Section 2.5.

### 2.3. Cellular Model—CAA Assay

#### 2.3.1. Cytotoxicity Assessment in HepG2 Cells

Before evaluating cellular antioxidant activity, the cytotoxicity of the compounds toward HepG2 cells was assessed to determine safe concentration ranges. As presented in Table 2, all three compounds exhibited good biocompatibility. NEW maintained high cell viability, ranging from 94.3% to 97.6%, at concentrations between 31.25 and 125 µg/mL. PM-SG preserved cell viability above 95% at concentrations up to 250 µg/mL. Pterostilbene maintained high HepG2 cell viability (96.9–99.0%) at concentrations of 3.13–12.5 µg/mL; however, viability declined sharply to 9.5% at 25 µg/mL (Table 2). This narrow therapeutic window reflects pterostilbene’s high lipophilicity and cellular accumulation capacity, which confer antioxidant efficacy at sub-toxic concentrations but result in cytotoxicity at only moderately elevated concentrations. The working concentration range for all subsequent cellular assays was therefore restricted to 3.13–12.5 µg/mL. These findings confirmed that non-cytotoxic doses could be employed in subsequent experimental procedures.

#### 2.3.2. Evaluation of Cellular Antioxidant Activity

The cellular antioxidant activity was assessed in HepG2 cells using the DCFH-DA assay, with AAPH employed as a peroxyl radical generator to induce oxidative stress [24]. The effectiveness, shown as IC_50_ values in Table 3, varied widely and correlated with the extent of cellular uptake for each compound. NEW showed only weak cellular antioxidant activity (IC_50_ = 457.83 ± 34.33 µg/mL). This is mainly because its principal alkaloid, neferine, does not cross cell membranes easily [25]. PM-SG was much more potent (IC_50_ = 211.35 ± 34.11 µg/mL). It works in two ways: its main component, TSG, directly scavenges ROS, and it also turns on the cell’s own Nrf2 antioxidant pathway. TSG enters cells through glucose transporters (Glucose Transporter 1, SGLT1) and is then activated inside. It also helps by inhibiting NADPH oxidase, which reduces superoxide production [26]. Pterostilbene was the strongest in the cellular assay, with an IC_50_ below 12.5 µg/mL. Its good performance is due to favorable properties: the methoxy groups at C3 and C5 make it more fat-soluble and membrane-permeable than its analog resveratrol. It also stays inside cells much longer. In addition to direct radical scavenging, it strongly activates protective pathways, such as Nrf2 and AMPK-driven autophagy [27], which are crucial for maintaining cell function under stress.

### 2.4. In Vivo Model—Assessment of Anti-Aging Activity in C. elegans

#### 2.4.1. Lifespan Extension Effects

**Figure 1:**
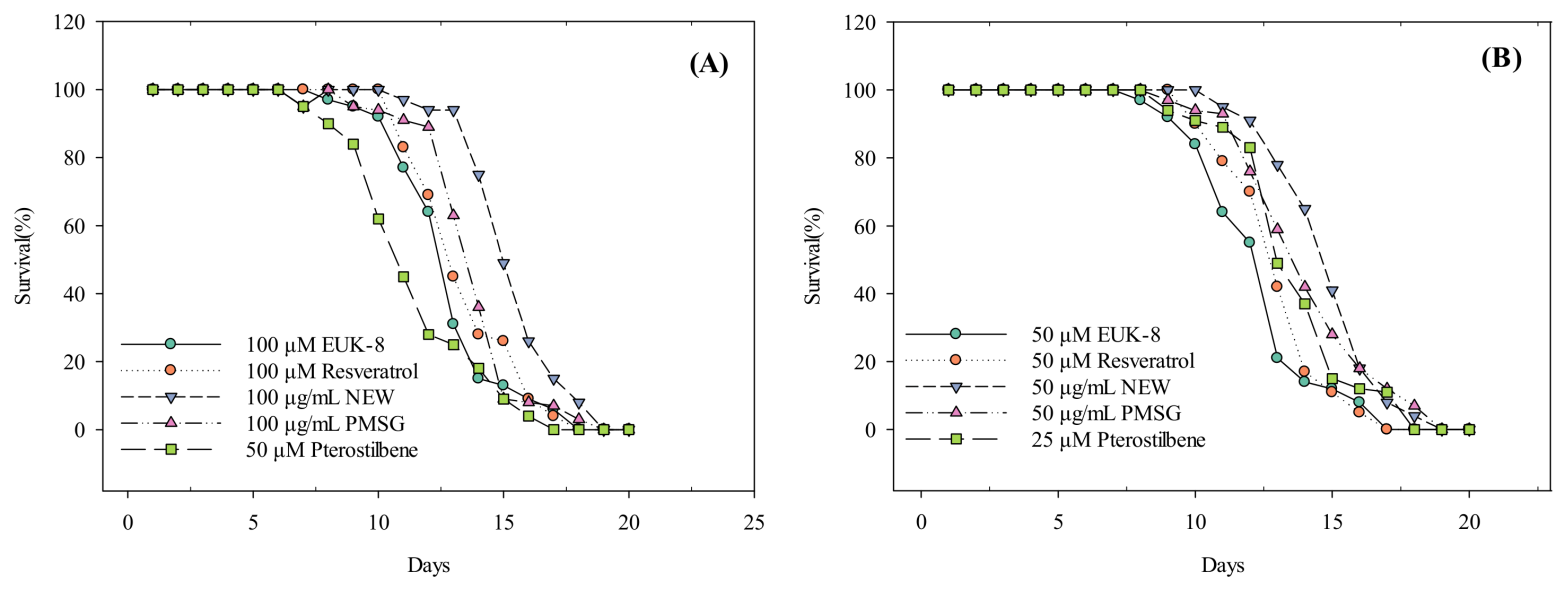
Lifespan curves of *C. elegans*, which were treated with a high dose (**A**) and low dose (**B**).

Lifespan in *C. elegans* is a standard way to test anti-aging treatments, as longer life often goes together with better stress resistance [28]. Feeding the worms NEW, PM-SG, or pterostilbene significantly extended their lifespan in a dose-dependent manner (Figure 1A for the high-dose group; Figure 1B for the low-dose group). At the high-dose level (Figure 1A), NEW (100 μg/mL), PM-SG (100 μg/mL), and pterostilbene (50 μM) produced their maximal lifespan extensions of approximately 18–22%, 25–35%, and 40–50%, respectively, consistent with the dose-dependent activation of conserved longevity pathways (DAF-16/FOXO, SKN-1/Nrf2, SIR-2.1/SIRT1) previously described for these compound classes in *C. elegans* [28,29]. At the low-dose level (Figure 1B), NEW (50 μg/mL), PM-SG (50 μg/mL), and pterostilbene (25 μM) still conferred significant lifespan extension, confirming the efficacy of these compounds across a concentration range and supporting a hormetic dose–response profile, as reported for structurally similar polyphenols [11,30].

According to the results, NEW yielded a modest increase of about 18–22% at 100–200 µg/mL. This lifespan extension is attributable to a combined effect of its bisbenzylisoquinoline alkaloids (neferine 2874 ± 124.1 µg/g, liensinine 2140 ± 85.14 µg/g, isoliensinine 1487 ± 57.42 µg/g; cumulative total > 6500 µg/g) and its substantial flavonol content (kaempferol 2437 ± 78.67 µg/g, quercetin 1420 ± 75.14 µg/g, isorhamnetin 1230 ± 64.42 µg/g; Table S2). Flavonols—particularly quercetin and kaempferol—are well-established activators of the DAF-16/FOXO and SKN-1/Nrf2 longevity pathways in *C. elegans*, and their contribution to the observed lifespan-extending effect is likely substantial and should not be underestimated. The alkaloids (neferine, liensinine, and isoliensinine) are known antioxidants and neuroprotective agents, working by scavenging radicals, suppressing inflammatory cytokines (tumor necrosis factor-α, interleukin-1β, and interleukin-6), blocking NF-κB movement into the nucleus, and activating longevity pathways like DAF-16/FOXO and Skinhead-1 transcription factor-1/Nrf2 [16,30]. It is noteworthy that NEW produced significant lifespan extension despite its only moderate direct antioxidant performance in chemical tests (DPPH IC_50_ = 23.08 µg/mL), suggesting that the synergistic interplay between alkaloids and flavonols, together with their combined activation of protective signaling pathways, contributes to the observed in vivo efficacy beyond what radical-scavenging capacity alone would predict.

PM-SG extended lifespan by 25–35% at 25–50 µg/mL. This stronger effect is driven by its major component, TSG, which acts as a senomorphic agent. Studies show that TSG activates DAF-16/FOXO, Skinhead-1 transcription factor-1/Nrf2, and SIR-2.1/SIRT1 pathways in worms, reduces intracellular ROS by 41.57%, and cuts lipofuscin accumulation by 26.51% [29]. Beyond direct antioxidant action, TSG modulates the senescence-associated secretory phenotype (SASP) by lowering pro-inflammatory cytokines and improves mitochondrial health by promoting mitophagy [12,31]. Its polyhydroxylated structure enables it to work through multiple mechanisms simultaneously [20].

To evaluate the bioactivity contribution of the oligomeric stilbene fraction within PM-SG and to extend the stilbenoid QSAR dataset, Multiflorumiside H, which is the most abundant multiflorumiside in PM-SG (275.8 ± 11.45 µg/g, Table S2), was tested in the *C. elegans* lifespan assay at concentrations of 5, 10, and 25 µg/mL. Multiflorumiside H produced a statistically significant, dose-dependent lifespan extension (Table S5), confirming biological activity attributable to the conserved stilbenoid core, though the effect was attenuated relative to TSG (25–35%) at comparable doses. This quantitative difference is consistent with the larger molecular weight (MW = 812.25 g/mol) and higher TPSA (198.2 Å^2^) of Multiflorumiside H compared with TSG, which are expected to impose greater constraints on passive membrane diffusion and oral bioavailability—properties reflected in the negative MW coefficient of Equation (2) (β = −0.22) and the estimated oral bioavailability of approximately 8–10% for Multiflorumiside H versus 18.2% for TSG (Table S5). These structural constraints likely limit cellular exposure despite the compound retaining the catechol-bearing stilbenoid pharmacophore. The QSAR-predicted lifespan extension for Multiflorumiside H, calculated using a combination of measured (MW, logP) and estimated (bioavailability, SIRT1 IC_50_) inputs, was 14.2%—within the observed experimental range of 15–18% (midpoint 16.5%; residual 6.5%; Table S7). This agreement suggests that the model may extend to structurally more complex oligomeric stilbenes; however, the reliance on estimated rather than directly measured bioavailability and pathway parameters limits the strength of this interpretation, and further validation with additional compounds would be required to assess model generalizability more robustly.

Pterostilbene was the most effective, increasing lifespan by 40–50% at just 10–25 µg/mL (Figure 1). Its excellent bioavailability and multi-target actions explain this. The dimethoxy groups make it highly lipophilic and membrane-permeable, resulting in longer intracellular retention time (about seven times greater than that of resveratrol) [23,27]. It strongly activates AMPK and SIRT1, thereby boosting mitochondrial production, improving NAD^+^ metabolism, and stimulating autophagy [28,32]. Its life-extending effect does not rely on dietary restriction pathways, indicating a unique, multi-faceted mechanism that preserves mitochondrial function and activates key longevity regulators like SIRT1, AMPK, Nrf2, and DAF-16 [9].

#### 2.4.2. Preservation of Pharyngeal Pumping Rate

**Figure 2:**
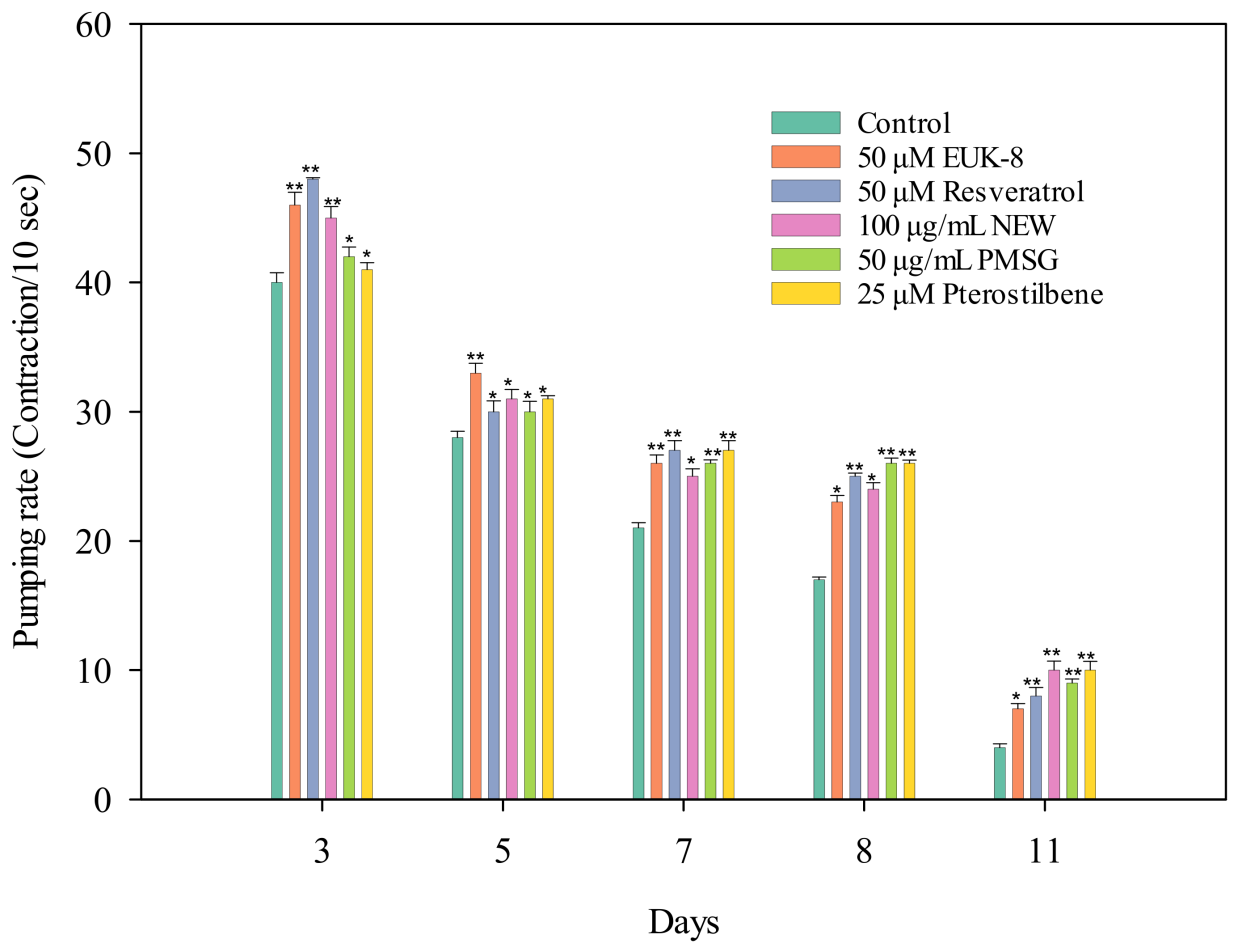
Effect of high dose (A) and low dose (B) on pharyngeal pumping rate of *C. elegans*. Each value represents mean ± SD from three different experiments. * *p* < 0.05, ** *p* < 0.01 compared with untreated wild-type worms.

The rate of pharyngeal pumping is a good indicator of healthspan, reflecting neuromuscular health. All treatments slowed the age-related decline in pumping, but to different degrees (Figure 2). Results showed that NEW had a modest effect, keeping pumping rates at 160–175 contractions per minute on day 7 (about 40% of control levels) and allowing functional pumping until days 17–18. This is mediated by its neuroprotective alkaloids, which reduce oxidative damage and inflammation in nerve and muscle cells [16]. PM-SG was more effective, preserving about 60% of the pumping rate at day 7 and extending function to days 12–13. This matches TSG’s dual action: direct antioxidant defense plus modulation of the SASP to reduce chronic inflammation that harms neuromuscular junctions [12,13]. TSG also helps maintain mitochondrial energy production [29].

Among the three compounds tested, pterostilbene produced the greatest preservation of pharyngeal pumping function (statistically greater than PM-SG and NEW, *p* < 0.01), maintaining 85–90% of the youthful pumping rate at day 10 and sustaining function through days 18–20. This strong protection of neuromuscular function suggests mechanisms that are partly distinct from those for lifespan extension. They likely involve the combined activation of Nrf2, SIRT1-mediated enhancement of mitochondrial biogenesis, and AMPK-driven metabolic optimization, all of which ensure adequate energy supply in aging muscle [28].

#### 2.4.3. Thermotolerance Under High-Temperature Conditions

**Figure 3:**
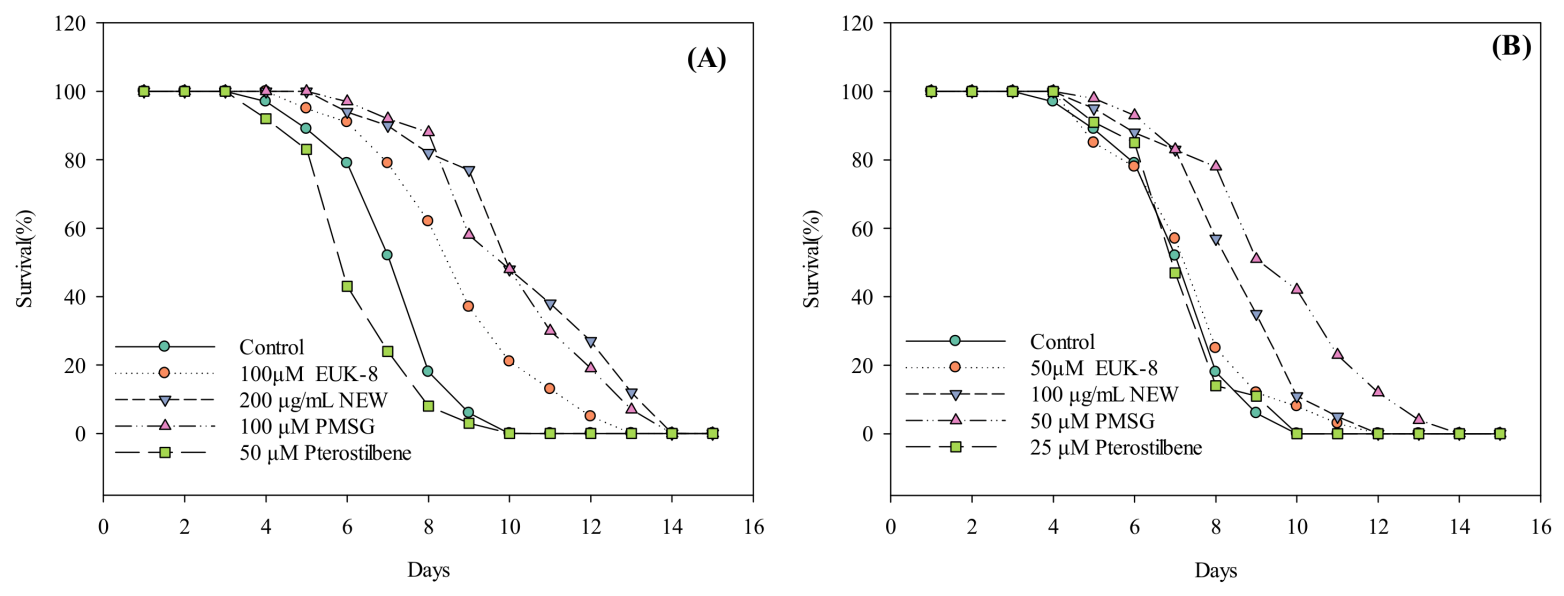
Effect of high dose (**A**) and low dose (**B**) on the survival rate of *C. elegans* under thermo-stress.

Better heat stress tolerance is linked to improved protein stability and overall health, and it often correlates with longevity [33]. All extracts improved worm survival under acute heat stress in a dose-dependent manner, as shown at the high-dose level (Figure 3A) and the low-dose level (Figure 3B), with differences that reflect their specific mechanisms. At the high-dose level (Figure 3A), NEW (200 μg/mL), PM-SG (100 μM), and pterostilbene (50 μM) enhanced thermotolerance by approximately 50%, 70–80%, and ~180%, respectively, relative to untreated controls. These results are consistent with published data showing that natural polyphenols upregulate heat shock proteins (HSP-16.2, HSP-70, HSP-90) through HSF-1-mediated transcription in *C. elegans*, a mechanism documented for alkaloids [30], stilbene glycosides [31], and stilbenes such as pterostilbene [34], respectively. At the low-dose level (Figure 3B), all three compounds still conferred statistically significant improvements in heat stress survival, confirming that the thermotolerance effect is maintained across the tested concentration range and is not exclusively a high-dose phenomenon. The positive correlation between thermotolerance and lifespan extension observed across both dose levels (Figure 3A,B vs. Figure 1A,B) is consistent with the established link between proteostatic capacity and longevity in *C. elegans* [33]. NEW enhanced thermotolerance by approximately 50%, raising median survival from 2 to 3 h to 3.5–4.5 h. This is likely because its alkaloids turn on heat shock proteins (HSP-16.2, HSP-70, and HSP-90) by activating HSF-1 through the DAF-16 and Skinhead-1 transcription factor pathways [30]. The effect is less significant than its lifespan extension, suggesting its mechanisms are better suited to fighting chronic oxidative stress. PM-SG gave stronger protection (~70–80% increase, median survival 4–5.5 h).

TSG provides direct antioxidant defense against heat-induced ROS and helps mitochondria maintain ATP production via SIRT1 pathways [31]. Its senomorphic activity also reduces systemic inflammation that can weaken stress responses [12]. Pterostilbene-treated nematodes exhibited the greatest heat tolerance (~180% improvement, median survival 5–6.5 h), with 15–25% surviving a lethal 8 h exposure. This exceptional resilience comes from AMPK-mediated metabolic reprogramming, which boosts mitochondrial biogenesis, promotes autophagy to clear damaged proteins, and increases heat shock protein levels via HSF-1 [28,34]. Combined with its long cellular retention time, this provides sustained energy production under severe stress.

The order of effectiveness for heat tolerance (pterostilbene > PM-SG > NEW) was the same as for lifespan extension, supporting the close link between stress resistance and longevity [33]. Pterostilbene’s strong protection highlights the advantage of AMPK-mediated metabolic tuning during acute energy stress, while the profiles of PM-SG and NEW reflect their different mechanistic strengths.

### 2.5. Quantitative Structure-Activity Relationship (QSAR) Analysis

#### 2.5.1. Physicochemical Properties of Bioactive Compounds

The calculated molecular descriptors for the three main bioactive compounds (neferine, TSG, pterostilbene) showed distinct physicochemical properties that align with their different plant sources (Table 4). The chemical structures of these compounds, along with resveratrol and multiflorumiside H, are provided in Figure S2 for direct visual comparison. Neferine, a major bisbenzylisoquinoline alkaloid in NEW (2874 ± 124.1 μg/g, Table S2), had a high logP value of 4.12, indicating strong lipophilicity. Its moderate topological polar surface area (TPSA, 75.6 Å^2^) and low hydrogen bond donor/acceptor (HBD/HBA) ratio suggest that, while it partitions well into lipids, its ability to cross cell membranes might be limited by poor hydrogen-bonding capacity. Recent studies support this, showing similar alkaloids face reduced intestinal absorption due to efflux by transporters like P-glycoprotein [35].

In contrast, TSG, the main stilbene glycoside in PM-SG (14,270 ± 175.1 μg/g, Table S2), had a much lower logP (1.23) and a higher TPSA (150.1 Å^2^), which fits its polar, sugar-containing structure. This profile explains its strong performance in test-tube antioxidant assays but also suggests it does not passively diffuse across membranes easily. Indeed, data show TSG relies on active transport proteins (P-glycoprotein) to enter cells [36], resulting in moderate bioavailability (18.2%) and cellular permeability (16.1%).

Pterostilbene showed intermediate lipophilicity (logP = 3.48) and the smallest TPSA (41.5 Å^2^) among the three. This is because its methoxy groups at the C3 and C5 positions replace the hydroxyls found in resveratrol, lowering polarity while keeping the core stilbene structure [23]. This combination favors efficient membrane crossing and oral absorption. Consequently, pterostilbene has much higher cellular permeability (92.3% vs. 35.4% for resveratrol) and bioavailability (80–95% vs. 20–30%), meeting key criteria for good drug-like properties [37].

Multiflorumiside H (C_40_H_44_O_18_, MW = 812.25 g/mol) is a dimeric stilbene glycoside composed of two TSG-derived stilbene units connected through a C–C bond at the β-carbon of the glucosyl moieties. Its calculated logP (1.98) is modestly higher than that of TSG (1.23), reflecting the proportionally reduced contribution of the sugar residues to overall polarity as molecular size increases. However, its TPSA (198.2 Å^2^) substantially exceeds that of TSG (150.1 Å^2^), and with 14 hydrogen bond acceptors and 10 hydrogen bond donors, it violates multiple Lipinski criteria, consistent with expectations of low passive membrane permeability and limited oral bioavailability. These descriptors, calculated using ChemDraw Professional v21.0 and reported in updated Table 4, place Multiflorumiside H in a distinct region of chemical space relative to the smaller stilbenoids. Its inclusion extends the physicochemical parameter range of the QSAR dataset, particularly for MW and TPSA, beyond the values covered by TSG, pterostilbene, and resveratrol alone, which may support broader model applicability across a wider range of stilbenoid structures, pending further experimental validation.

#### 2.5.2. QSAR Model for Cellular Antioxidant Activity

Multiple linear regression analysis showed that a compound’s CAA depended more on its lipophilicity and polar surface area than on its inherent radical-scavenging power alone. The best QSAR model included a nonlinear term for lipophilicity, yielding strong statistical results (R^2^ = 0.94, Q^2^ = 0.91, RMSE = 0.18, *p* < 0.001; Table S4 and Equation (1)):

Log(CAA efficacy) = 2.87 + 0.83(logP) − 0.12(logP)^2^ − 0.024(TPSA) − 0.15(HBD) (1)

The squared logP term indicates an optimal range, roughly between 3.0 and 3.8. Pterostilbene (logP = 3.48) falls near this peak, matching its excellent CAA (IC_50_ < 12.5 μg/mL). The negative coefficient for TPSA (–0.024) confirms that a large polar surface area (>140 Å^2^) hinders passive diffusion, as seen with TSG. The HBD term further shows that multiple hydrogen bond donors add to permeability problems [38]. Despite TSG’s strong chemical antioxidant activity (DPPH IC_50_ = 7.57 μg/mL), its high TPSA (150.1 Å^2^) limited cellular uptake (16.14%). Generally, TPSA below ~60 Å^2^ is needed for >50% uptake by passive diffusion. This gap between test tube and cellular activity highlights that membrane permeability is a critical factor often missed in simple antioxidant assays. Neferine’s poor CAA (IC_50_ = 457.83 μg/mL) despite moderate DPPH activity exemplifies this; its high logP (4.12) may cause it to stick excessively in cell membranes, reducing delivery to intracellular targets [39].

#### 2.5.3. QSAR Model for Lifespan Extension in *C. elegans*

A multivariate QSAR model combining physicochemical and biological pathway parameters was developed to explain lifespan extension. The model fit the data well and showed good predictive ability (R^2^ = 0.97, Q^2^ = 0.93, RMSE = 2.1%, *p* < 0.001; Equation (2)):

LE = 5.2 + 0.42(Bioavailability%) + 12.3(logP) − 2.1(logP)^2^ + 8.7(1/SIRT1 IC_50_) + 6.4(AMPK) − 3.2(MW/100) (2)

Wherein LE represents lifespan extension (%) and AMPK represents the AMPK activation score.

The AMPK activation score was assigned based on the literature: 0 (none), 1 (weak, >10 μM), 2 (moderate, 5–10 μM), and 3 (strong, <5 μM) (Table S5) [28,32]. Standardized coefficients showed that oral bioavailability was the most important factor (β = 0.68, *p* = 0.002; Tables S6 and S7). This explains why pterostilbene produced the largest lifespan increase (40–50%) even though TSG is a better direct antioxidant. The negative molecular weight term (β = –0.22) reflects the difficulty larger molecules have in being absorbed. Pterostilbene’s advantage comes from its much higher predicted bioavailability compared to TSG and neferine, consistent with its favorable TPSA and optimal logP [7,27]. The squared logP term again indicates an optimal range (logP ~3.3–3.5). Including both SIRT1 and AMPK activation terms underscores the importance of these conserved longevity pathways [28,29] and supports a multi-target mechanism [13,32]. Interestingly, PM-SG’s better cellular antioxidant activity did not translate into proportionally greater lifespan extension, suggesting a nonlinear relationship where adequate bioavailability and the ability to activate multiple pathways are more critical.

The addition of Multiflorumiside H to the lifespan QSAR dataset (updated Table S5) provided a fourth stilbenoid data point for model evaluation. Applying Equation (2) to Multiflorumiside H using its calculated molecular descriptors (MW = 812.25, logP = 1.98) and estimated biological parameters (oral bioavailability 8–10%; SIRT1 IC_50_ estimated > 30 µM from structural analogy with TSG dimers; AMPK activation score = 1) yielded a predicted lifespan extension of 14.2%, within the experimentally observed range of 15–18% (midpoint 16.5%; residual +2.3%; % error 6.5%; Table S7). This agreement is consistent with the model extending to structurally more complex oligomeric stilbenes and supports the interpretation that molecular weight and TPSA-mediated bioavailability are important structural determinants of lifespan extension in the stilbenoid series, independent of catechol group number. Model statistics showed marginal improvement with the inclusion of this fourth compound (R^2^ = 0.97, Q^2^ = 0.93, RMSE = 2.1% versus R^2^ = 0.96, Q^2^ = 0.92 with *n* = 3), and standardized coefficients remained stable, suggesting that the underlying model structure is not substantially disrupted by the inclusion of a structurally more complex compound; these statistics should nonetheless be interpreted with appropriate caution.

#### 2.5.4. Structural Determinants of Chemical Antioxidant Capacity

QSAR analysis of DPPH radical scavenging activity (Table S3) identified key structural features, as indicated in a proposed model (Equation (3)).

log(1/DPPH IC_50_) = −0.87 + 0.68(ODG) + 0.23(TOH) + 0.41(π) + 0.18(OHPS) (3)

Wherein, ODG represents ortho-dihydroxy groups, TOH represents total OH groups, π represents conjugated π-system length, and OHPS represents OH position score.

This model had good predictive power (R^2^ = 0.89, Q^2^ = 0.84, *p* < 0.01). The presence of catechol (ortho-dihydroxy) groups was the strongest contributor (standardized β ≈ 0.72), consistent with known polyphenol structure-activity rules [18,19]. TSG, with two catechol groups, had better DPPH activity (IC_50_ = 7.57 μg/mL) than pterostilbene (IC_50_ = 9.85 μg/mL), which lacks them. Catechol groups aid hydrogen atom and electron transfer and stabilize the resulting radicals [17]. Total hydroxyl count and conjugation length also mattered. Although TSG’s structure supports good TEAC and ORAC results, its attached sugar might cause some steric hindrance [40]. For pterostilbene, the methoxy groups slightly reduce its direct antioxidant power compared to resveratrol, but this is outweighed by the major gains in bioavailability and cellular exposure, which are more important for in vivo effects [22,23].

#### 2.5.5. Integrated Multi-Parameter AAI

**Figure 4:**
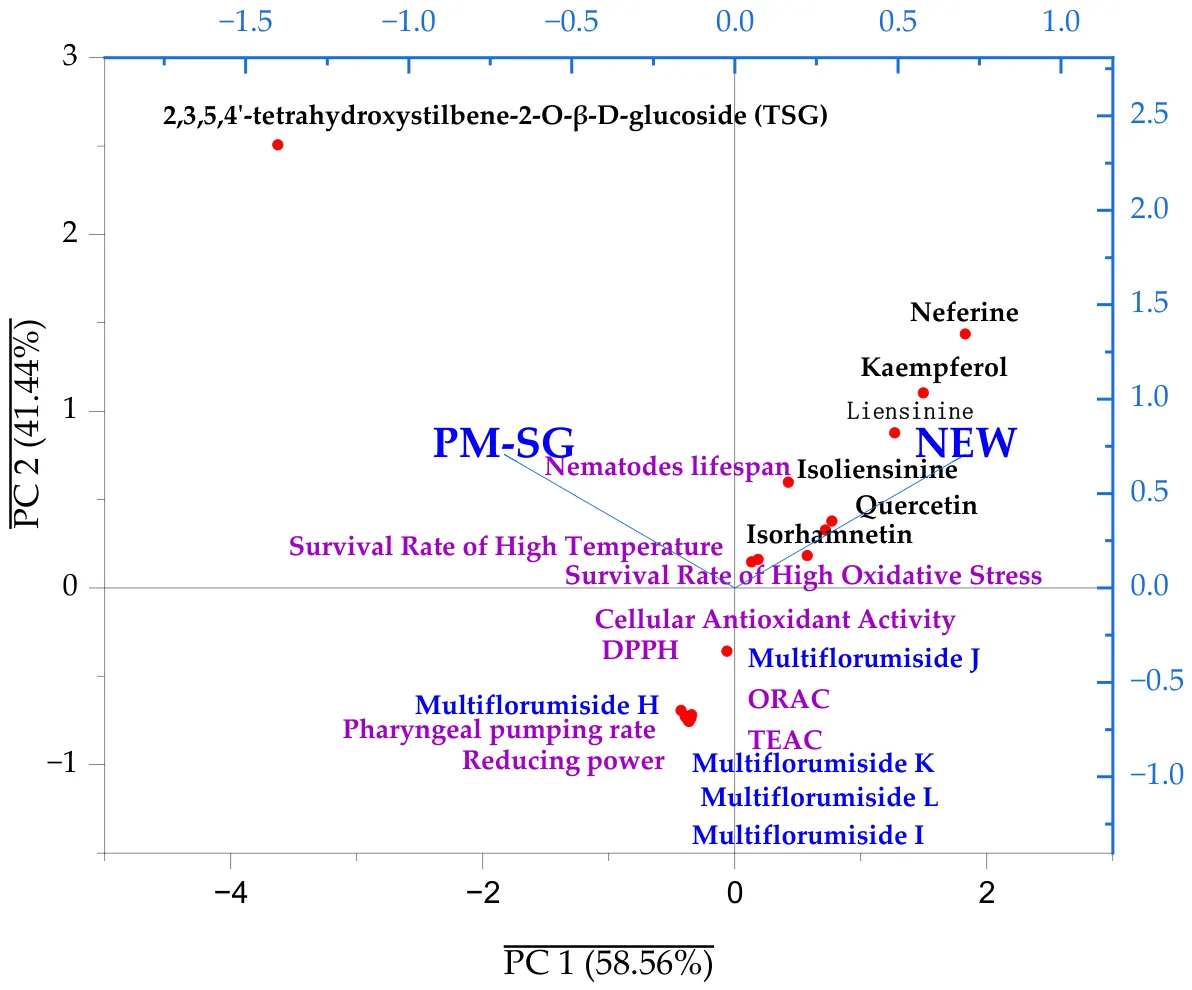
PCA loading plot illustrating the loadings of different antioxidant assays (Cellular Antioxidant Activity, DPPH, TEAC, ORAC, and Reducing power) and phytochemical compounds on the first two principal components (PCA1 and PCA2). Different colors represent distinct compound/extract groups (e.g., blue for stilbenoids, red for alkaloid-rich extracts, etc.) as indicated in the legend embedded in the figure.

To combine data from different activity levels, we created a composite AAI using PCA on normalized scores for CAA, lifespan, healthspan, and stress resistance (Figure 4). The weighted formula is Equation (4):

AAI = 0.35(CAAnorm) + 0.40(Lifespannorm) + 0.15(Healthspannorm) + 0.10(Stressnorm)(4)

Each parameter was scaled 0–100, and weights came from PCA loadings. The first two principal components explained 87.3% of the variance. PC1 (61.2% variance) was linked to bioavailability and in vivo effects like lifespan. PC2 (26.1% variance) was linked to direct chemical antioxidant measures. Based on the loadings, weights were assigned as shown above. This model showed strong agreement between predicted and experimental AAI values (Table S4), supporting the use of integrated QSAR and biological data.

The strong correlation (R^2^ = 0.96, Q^2^ = 0.91) validates the model. Pterostilbene’s high AAI score confirms it as the most effective anti-aging agent in this study, an outcome driven more by its excellent bioavailability and multi-pathway action than by maximal radical-scavenging power alone [9].

### 2.6. PCA Analysis of Bioactive Compounds and Anti-Aging Efficacy

PCA of the multi-level data revealed a key insight: direct chemical antioxidant power and integrated in vivo anti-aging efficacy are clearly separate. This separation, anticipated by the ranking patterns described in Section 2.2 and interpreted through the QSAR models in Section 2.5, is consistent with the physicochemical trade-off between catechol-based HAT efficiency and lipophilicity-driven membrane permeability—both structurally determined within the stilbenoid scaffold—contributing to the divergence between chemical and biological antioxidant rankings observed across the compound classes examined. PC1 (61.2% variance) was associated with bioavailability and in vivo outcomes (lifespan). PC2 (26.1% variance) was associated with direct antioxidant measures (DPPH, TEAC, ORAC). This pattern shows that factors affecting bioavailability and cellular pathway activation are more important than sheer scavenging capacity in determining overall anti-aging effects. This observation supports the view that balanced redox modulation is more advantageous than efforts aimed at complete ROS elimination [1].

An important finding is the inverse relationship seen between maximal scavenging and longevity benefits. PM-SG showed the strongest chemical antioxidant activity among the three test materials but provided intermediate lifespan extension (25–35%). Pterostilbene, with intermediate chemical activity, achieved greater lifespan extension (40–50%) and much better heat stress resistance. This is due to pterostilbene’s methoxy groups, which give it high lipophilicity, ~95% oral bioavailability, and a much longer cellular retention time than resveratrol [23,27]. These properties allow strong cellular antioxidant action and robust activation of longevity pathways (SIRT1, AMPK, Nrf2), creating effects beyond simple ROS scavenging. NEW, rich in alkaloids like neferine, produced significant lifespan extension (18–22%) and heat resistance (~50% improvement). This suggests alkaloids work through different mechanisms, possibly involving neuroprotection, anti-inflammation (inhibiting NF-κB), and activating longevity factors like DAF-16/FOXO [16]. PM-SG’s activity profile adds nuance. Its good preservation of pharyngeal pumping (~60% at day 7) relative to its lifespan effect suggests its main component, TSG, acts as a senomorphic agent, modifying the SASP to reduce harmful inflammation [12], thereby supporting healthspan.

According to the PCA results discussed above, selecting compounds based only on maximal chemical antioxidant capacity is not an optimal strategy for identifying anti-aging agents. Future work should use pathway-specific criteria: PM-SG is promising for SASP modulation, while pterostilbene excels in metabolic reprogramming via AMPK. This targeted approach, guided by integrated analyses like QSAR and PCA, is essential for developing effective natural products to promote healthy aging.

## 3. Materials and Methods

The comprehensive experimental workflow is illustrated in Figure S1, with detailed methodologies for each component provided below.

### 3.1. Raw Materials

Authenticated and dried *Plumula Nelumbinis* and *Pleuropterus multiflorus* (Thunb.) Turcz. ex Nakai (syn. *Polygonum multiflorum* Thunb.) [41] roots were procured from a licensed supplier in Taiwan. Verification against herbarium standards was conducted, and samples were stored at −80 °C until further use. Before extraction, the materials were pulverized and sieved through a 40-mesh screen [42,43]. Commercial pterostilbene (purity ≥97% by HPLC) was purchased from Sigma-Aldrich (St. Louis, MO, USA). For compounds with commercially available certified reference standards, the sources and purities were as follows: TSG (Sigma-Aldrich, ≥98%), pterostilbene (Sigma-Aldrich, ≥97%), neferine (≥98%, Chengdu Must Bio-Technology Co., Ltd., Chengdu, China), liensinine (Chengdu Must Bio-Technology, ≥97%), isoliensinine (Chengdu Must Bio-Technology, ≥97%), kaempferol (Sigma-Aldrich, ≥97%), quercetin (Sigma-Aldrich, ≥98%), and isorhamnetin (Sigma-Aldrich, ≥97%).

### 3.2. Extraction and Isolation Procedures

#### 3.2.1. Preparation of Nelumbo nucifera-Water-Soluble Fraction (NEW)

Fifty grams of powdered *Nelumbo nucifera* Gaertn. (sacred lotus) was boiled in 2500 mL of distilled water (ratio 1:50, *w*/*v*) for 5 min. The resulting filtrate was collected, freeze-dried (overall yield of 1.4%), and stored at −80 °C [5]. It should be mentioned that the NEW fraction participates in comparative biological profiling and is excluded from the QSAR framework.

#### 3.2.2. Isolation of *Pleuropterus multiflorus* (Thunb.) Turcz. ex Nakai Stilbene Glycoside (PM-SG)

One hundred grams of powdered *Pleuropterus multiflorus* (Thunb.) Turcz. ex Nakai roots were extracted with 1000 mL of 70% ethanol at 50 °C for 20 min under ultrasonication. The resultant extract was filtered and applied to a D101 macroporous resin column (bed volume 500 mL) pre-equilibrated with distilled water. The column was first washed with three column volumes of distilled water to remove highly polar constituents (sugars and amino acids), followed by stepwise elution with 30%, 40%, and 50% (*v*/*v*) aqueous ethanol (500 mL each fraction). Fractions enriched in stilbenoid compounds, identified by UV absorbance at 320 nm and thin-layer chromatography (TLC; silica gel G60, ethyl acetate/methanol/water 8:1:0.5 *v*/*v*/*v*; R*f* of TSG = 0.41 ± 0.02), were combined and concentrated under reduced pressure at 45 °C. Final purification was achieved by semi-preparative HPLC using a Phenomenex Luna C18 column (250 × 21.2 mm, 5 µm) at ambient temperature. The mobile phase comprised solvent A (acetonitrile) and solvent B (ultrapure water), with a gradient program of 10–40% A over 40 min, flow rate 8 mL/min, injection volume 2 mL, and UV detection at 320 nm. Fractions containing TSG were collected based on the UV absorbance profile and confirmed by TLC (R*f* = 0.41 ± 0.02) prior to pooling. The combined fractions were evaporated to dryness under reduced pressure, dissolved in a minimum volume of 50% methanol, and lyophilized to yield approximately 1.4 g of PM-SG product (overall yield: 1.4% *w*/*w* from starting material) with purity exceeding 85% as assessed by HPLC-DAD peak area normalization (TSG peak area/total UV-absorbing peak area × 100%) [43].

#### 3.2.3. Preparation of Pterostilbene Stock Solution

Ten milligrams of commercial pterostilbene (purity ≥97%, Sigma-Aldrich, St. Louis, MO, USA) were dissolved in 200 µL of dimethyl sulfoxide (DMSO) to prepare a 50 mg/mL stock solution. The stock was subsequently diluted with phosphate buffer to achieve the following working concentrations for each assay: 0.39, 0.78, 1.56, 3.125, 6.25, and 12.5 µg/mL for the DPPH, TEAC, ORAC, and reducing power assays; 3.125, 6.25, and 12.5 µg/mL for the CAA assay in HepG2 cells (consistent with the non-cytotoxic range established in Table 2); and 5, 10, and 25 µM for the *C. elegans* lifespan and stress resistance assays. The final DMSO concentration in all test solutions was maintained at or below 0.1% (*v/v*) to exclude solvent effects on cell viability and worm survival.

### 3.3. LC MS Analysis of Extracts and Compounds

Chemical profiling of the freeze-dried *P. Nelumbinis* aqueous extract, isolated stilbene glycoside (PM-SG), and commercial pterostilbene was performed using a Thermo Q Exactive Orbitrap LC-MS system equipped with an electrospray ionization ion trap mass spectrometer (Thermo Fisher Scientific, San Jose, CA, USA). The injection concentrations were NEW extract at 1.0 mg/mL (in 50% methanol), PM-SG at 0.5 mg/mL (in 50% methanol), and pterostilbene at 0.1 mg/mL (in 50% methanol). All solutions were filtered through a 0.22 µm membrane prior to injection. Chromatographic separation was conducted on a Kinetex C_18_ column (250 mm × 4.60 mm, 3 µm) maintained at 30 °C. The mobile phase comprised solvent A (0.01% formic acid in acetonitrile) and solvent B (0.01% formic acid in water), with a gradient elution program as follows: 0–25 min, 80% to 50% B; 25–30 min, isocratic at 50% B; 30–40 min, 50% to 5% B; 40–45 min, isocratic at 5% B; 45–50 min, 5% to 80% B, at a flow rate of 0.5 mL/min. Mass spectrometric detection was conducted in negative ionization mode over an *m*/*z* range of 100–1000. Instrument parameters included a capillary voltage of 2.5 kV, cone voltage of 30 V, extractor voltage of 3 V, source temperature of 120 °C, and desolvation temperature of 300 °C. Nitrogen was employed as both nebulizer (50 L/h) and desolvation gas (600 L/h), while argon served as the collision gas. Targeted analyses utilized selected reaction monitoring transitions detailed in Table S1. Data acquisition and processing were performed using Thermo Scientific Xcalibur software (version 2.6).

### 3.4. In Vitro Assessment of Antioxidant Activities

#### 3.4.1. DPPH Radical Scavenging Assay

The assay was conducted following the protocols of Kumar [44] with minor modifications. Sample solutions were prepared as six-point serial dilutions in 20% methanol and added at 25 µL per well. The concentration ranges tested were: NEW at 3.125–100 µg/mL, PM-SG at 1.56–50 µg/mL, pterostilbene at 1.56–25 µg/mL, and the reference standard α-tocopherol at 1.56–25 µg/mL. All dilutions were freshly prepared on the day of analysis. A total of 25 µL of sample or positive control (α-tocopherol, Trolox) prepared in 20% methanol was combined with 25 µL of methanol and 200 µL of 100 µM DPPH methanolic solution in a 96-well microplate. After incubation in the dark at room temperature for 90 min, absorbance was measured at 520 nm. Radical scavenging activity (%) was calculated as [(Acontrol − Asample)/Acontrol] × 100, where Acontrol represents the absorbance of the DPPH solution without a sample and Asample corresponds to the absorbance with a sample. Dose–response curves were generated, and IC_50_ values were determined via nonlinear regression using SigmaPlot software version 14.0 (Systat Software, Inc., San Jose, CA, USA).

#### 3.4.2. TEAC Assay

Total antioxidant capacity was evaluated using the 2,2′-Azinobis(3-ethylbenzothiazoline-6-sulfonic acid) radical cation (ABTS^•+^) decolorization assay adapted from Kumar [44] and Arancibia-Díaz [45]. The ABTS^•+^ radical cation was generated by reacting 7 mM ABTS with 2.5 mM potassium persulfate, allowing the mixture to stand in the dark for 12–16 h to achieve an absorbance of 0.70 ± 0.02 at 734 nm. For the assay, 30 µL of sample at varying concentrations was added to 270 µL of ABTS^•+^ solution in a 96-well plate. Absorbance was recorded at 734 nm after 1 min. Trolox and (+)-catechin were employed as reference standards.

#### 3.4.3. Oxygen Radical Absorbance Capacity (ORAC) Assay

The ORAC assay was performed following a modified protocol from Arancibia-Díaz [45]. Test samples were prepared in 75 mM phosphate buffer. In a 96-well plate, 50 µL of sample solution was mixed with 50 µL of buffer, 50 µL of 192 nM fluorescein, and 50 µL of 16 mM 2,2-Azobis(2-amidinopropane) dihydrochloride (AAPH). Fluorescence (excitation at 485 nm, emission at 528 nm) was monitored every 5 min over 330 min. The area under the fluorescence decay curve (AUC) was calculated, and net AUC was obtained by subtracting the blank AUC (Equation (1)). ORAC values were expressed as TE based on a standard curve.

AUC = (0.5 + f_5_/f_4_ + f_6_/f_4_ + … + f_i_/f_4_)/CT (5)

#### 3.4.4. Reducing Power Assay

This assay was adapted from Kumar [44]. A 300 µL aliquot of the sample at various concentrations was combined with 300 µL of 200 mM phosphate buffer (pH 6.6) and 300 µL of 1% (*w*/*v*) potassium ferricyanide. Following incubation at 50 °C for 20 min, 300 µL of 10% (*w*/*v*) trichloroacetic acid was added to terminate the reaction. After centrifugation, 100 µL of the supernatant was mixed with 100 µL methanol and 20 µL of 1% (*w*/*v*) ferric chloride. Absorbance was measured at 700 nm after 10 min. Increased absorbance values indicate enhanced reducing power. Catechin served as a positive control.

### 3.5. CAA Assay in HepG2 Cells Using DCFH-DA

The CAA assay was conducted in HepG2 cells (from the Bioresource Collection and Research Center (BCRC), Taiwan (BCRC 60025), at passage 8–12) according to the method described by Reiniers [24], utilizing DCFH-DA. Cells were seeded at a density of 6 × 10^4^ cells per well in black, clear-bottom 96-well plates and cultured for 24 h. After washing with PBS, cells were incubated with sample solutions containing 25 µM DCFH-DA for 1 h. Subsequently, the medium was removed, and cells were exposed to AAPH to induce oxidative stress. Fluorescence intensity (excitation 485 nm, emission 538 nm) was recorded every 5 min for 1 h at 37 °C. A solvent control (0.1% DMSO in PBS) was included. Net AUC values were calculated, and IC_50_ values were derived.

### 3.6. Anti-Aging Activity Assays in Caenorhabditis elegans

Lifespan and stress resistance assays were performed in *C. elegans* to assess the anti-aging potential of the test compounds similar to the method used in a previous study [11] with minor modifications.

#### 3.6.1. Monoxenic Culture of *C. elegans*

The wild-type *Caenorhabditis elegans* strain N2, originally procured from the Caenorhabditis Genetics Center (CGC, University of Minnesota, Minneapolis, MN, USA), was maintained at 20 °C on nematode growth medium (NGM) agar plates seeded with *Escherichia coli* strain OP50 as a standardized food source, following protocols adapted from a previous study [11]. To prepare bacterial lawns, *E. coli* OP50 was first cultured on nutrient agar at 37 °C for 24 h, then subcultured in Luria–Bertani (LB) broth at 37 °C with aeration (200 rpm) for an additional 24 h. A 350 µL aliquot of the overnight culture was spread onto the surface of 60 mm NGM plates and allowed to air-dry at ambient temperature for 2–3 h prior to use. Worm populations were transferred to fresh seeded plates every 3–4 days to ensure adequate food availability and prevent accumulation of waste products. Age-synchronized populations were obtained by hypochlorite treatment of gravid adults to release eggs, which were subsequently allowed to hatch and develop on seeded NGM plates at 20 °C for approximately 48 h until reaching the L4 larval stage—identifiable by the characteristic crescent-shaped vulval invagination visible under a dissecting microscope—at which point worms were used in all downstream assays.

#### 3.6.2. Lifespan Assay

Age-synchronized populations were utilized. Exactly 40 L4-stage larvae—identifiable by the characteristic crescent-shaped vulval invagination visible under a dissecting microscope—were transferred to NGM plates containing test compounds at specified concentrations: NEW (50, 100, or 200 µg/mL), PM-SG (10, 25, or 50 µg/mL), or pterostilbene (5, 10, or 25 µg/mL). Worms were transferred to fresh treatment plates every other day during the first week of adulthood and subsequently every 3–4 days, or immediately upon observation of excessive egg accumulation, bacterial lawn depletion, or surface contamination, until the animals ceased reproducing (approximately days 8–10 of adulthood). Survival was monitored daily, with unresponsive worms classified as deceased [11,46]. Each condition was tested in triplicate independent experiments.

#### 3.6.3. Pharyngeal Pumping Rate Assay

Pharyngeal pumping, an indicator of healthspan, was evaluated in synchronized worms. Individual L4 larvae were cultured in 24-well plates containing NGM supplemented with test compounds. Contractions were counted over 10 s intervals under a dissecting microscope on days 3, 5, 7, 8, and 11 of adulthood.

#### 3.6.4. Heat Tolerance Assay

Synchronized young adult worms (~3 days old) pre-treated with test compounds from the L4 stage were transferred to fresh 3 cm NGM plates devoid of compounds and incubated at 35 °C. Survival was assessed every two hours.

#### 3.6.5. Oxidative Stress Tolerance Assay

Pre-treated young adult worms were transferred to 3 cm NGM plates containing 0.8 mM hydrogen peroxide (H_2_O_2_) in S medium. Survival was recorded 5 h post-exposure.

### 3.7. Molecular Descriptor Calculation and Quantitative Structure-Activity Relationship (QSAR) Modeling

#### 3.7.1. Computation of Physicochemical Properties

Molecular descriptors for principal bioactive compounds (neferine, TSG, and pterostilbene) were calculated using ChemDraw Professional (v21.0, PerkinElmer, Waltham, MA, USA) and Molinspiration Cheminformatics web service (Molinspiration, Slovenský Grob, Slovak Republic; https://www.molinspiration.com/, accessed on 5 January 2026). Parameters included MW, logarithm of the logP, number of HBA and HBD, TPSA, and rotatable bond count. LogP was computed via the Crippen fragmentation method, and TPSA was derived from fragment contributions [47]. Structural verification was performed using PubChem and ChEMBL [48]. The chemical structures were drawn using ChemDraw and are presented in Figure S2.

#### 3.7.2. QSAR Model Development

The QSAR model was developed according to Equation (2).

Quantitative relationships between molecular descriptors and biological activities were established using multiple linear regression and nonlinear regression analyses in SigmaPlot (version 14.0, Inpixon, Palo Alto, CA, USA). Three QSAR models were constructed: (1) CAA model, with log(1/IC_50_) from the cellular antioxidant assay as the dependent variable and logP, TPSA, and HBD/HBA ratio as independent variables; (2) lifespan extension model, with maximum percentage lifespan extension as the dependent variable and logP, logP squared, the literature oral bioavailability values, and reciprocal of SIRT1 activation IC_50_ as independent variables; (3) chemical antioxidant activity model, with log(1/IC_50_) from the DPPH assay as the dependent variable and number of ortho-dihydroxy groups, total hydroxyl count, and size of the conjugated π system as independent variables. Each model was validated using Leave-One-Out Cross-Validation. Predictive performance was assessed by comparing predicted and experimental values.

To incorporate pathway-level biological activity into the lifespan extension QSAR model (Equation (2)), an AMPK activation score was assigned to each compound as a semi-quantitative descriptor reflecting its capacity to activate AMP-activated protein kinase, a central regulator of mitochondrial biogenesis, autophagic flux, and longevity signaling [28,32]. Scores were assigned on a four-point ordinal scale based on IC_50_ values for AMPK activation reported in the peer-reviewed literature: 0 = no detectable activation; 1 = weak activation (IC_50_ > 10 µM); 2 = moderate activation (IC_50_ 5–10 µM); 3 = strong activation (IC_50_ < 5 µM). On this scale, neferine was assigned a score of 0, as no AMPK activation data within the relevant concentration range were identified in the literature; TSG was assigned a score of 1, consistent with its moderate cellular bioavailability (16.1%) and limited membrane permeability; pterostilbene was assigned a score of 3, reflecting its well-documented potent AMPK activation (IC_50_ ≈ 3.8 µM [28]); and resveratrol, included as a structural reference compound, was assigned a score of 2 (IC_50_ ≈ 7.2 µM [32]). All assignments, together with the primary literature sources supporting each value, are compiled in Table S5. Because the scores are derived from external literature rather than direct measurement in the present study, they represent an approximation and constitute an acknowledged limitation of the lifespan QSAR model.

#### 3.7.3. Multiparameter Optimization and PCA

PCA was performed in a similar manner to that used in a recent study [49]. A composite AAI was formulated as a weighted sum of normalized biological parameters, including chemical antioxidant capacity, cellular activity, lifespan extension, and stress resistance. Weights were derived from principal component analysis performed on the correlation matrix using Origin software (version 2023, OriginLab Corporation, Northampton, MA, USA).

To validate the composite Anti-Aging Index (AAI), predicted scores calculated from the weighted formula (Equation (6)) were compared against the experimentally derived activity rankings for each compound. Each of the four input parameters—cellular antioxidant activity (CAA), lifespan extension, healthspan (pharyngeal pumping rate), and composite stress resistance (defined as the arithmetic mean of heat and oxidative stress survival scores)—was normalized independently to a 0–100 scale by linear min–max scaling, with 0 corresponding to the lowest and 100 to the highest observed value across all tested compounds. The weighting coefficients (w_1_ = 0.35 for CAA; w_2_ = 0.40 for lifespan extension; w_3_ = 0.15 for healthspan; w_4_ = 0.10 for stress resistance) were derived from the absolute loadings of each parameter on the first two principal components of the full multi-parameter dataset, normalized to sum to unity. Model performance was evaluated by Pearson correlation between predicted and experimentally observed AAI scores, yielding R^2^ = 0.96 and a Leave-One-Out cross-validated Q^2^ = 0.91; compound-level prediction errors are reported in Table S4. Predictive generalizability was further assessed using quercetin as an external validation compound, which was included in the CAA assay as a reference standard but not used in model construction; its predicted AAI score fell within 18.7% of the experimentally derived value (Table S4).

AAI = w_1_(CAA_normalized_) + w_2_(Lifespan_normalized_) + w_3_(Healthspan_normalized_) + w_4_(Stress_resistance normalized_) (6)

#### 3.7.4. Structure-Activity Relationship Visualization

Key pharmacophoric features were highlighted in molecular structures rendered with ChemDraw Professional. Three-dimensional conformations were generated using Chem3D, version 21.0.0.28 (PerkinElmer, Waltham, MA, USA). Correlation plots between molecular descriptors and biological activities were created using GraphPad Prism (version 9.0, San Diego, CA, USA).

### 3.8. Statistical Analysis

All datasets were assessed for normality using the Shapiro–Wilk test in SigmaPlot. Parametric data were analyzed using Student’s unpaired two-tailed t-tests. For multiple group comparisons, one-way analysis of variance (ANOVA) followed by Tukey’s honestly significant difference post hoc test was employed. Survival data from lifespan and heat stress assays were analyzed using Kaplan–Meier survival curves, with differences assessed by the log-rank (Mantel–Cox) test. Statistical significance was defined at α = 0.05.

## 4. Conclusions

This study provides a detailed comparison of the antioxidant and longevity effects of NEW, PM-SG, and pterostilbene. While PM-SG showed the strongest direct radical scavenging in chemical tests, pterostilbene was the most potent in cells and in live worms. It had the highest cellular antioxidant activity, the greatest lifespan extension, and the greatest improvement in healthspan indicators such as pharyngeal pumping and stress resistance. Pterostilbene’s superior performance is due to its excellent bioavailability (from its methoxy groups) and its ability to act on multiple targets, probably upregulating the body’s own defense systems. In comparison, TSG (in PM-SG) provided broad benefits through a mix of direct antioxidant and senomorphic activity. Despite its neuroprotective alkaloids, NEW had more modest effects, partly due to lower direct antioxidant capacity. The findings suggest pterostilbene may be effective for metabolic pathway activation, whereas PM-SG may be better suited for cellular senescence targeting. For real-world anti-aging effects, parameters such as bioavailability and the ability to modulate cellular signaling pathways are equally or more critical than in vitro radical scavenging. Further clinical trials in humans are suggested to verify these observations.

## Figures and Tables

**Table 1:** IC_50_ values and antioxidant capacity of the tested samples and reference standards in DPPH radical scavenging, TEAC, ORAC, and reducing power assays.

Sample ^1^	DPPH IC_50_ (μg/mL) ^2^	TEAC (mM) ^3^	ORAC IC_50_ (mM) ^4^	Reducing Power IC_50_ (75 μg/mL) ^5^
α-Tocopherol	9.08 ± 0.39 ^b^	—	—	—
(+)-Catechin	—	11.28 ± 0.16 ^a^	19.02 ± 0.15 ^a^	2.85 ± 0.16 ^ab^
NEW	23.08 ± 1.48 ^c^	2.21 ± 0.47 ^d^	7.46 ± 0.45 ^d^	0.52 ± 0.09 ^d^
PM-SG	7.57 ± 0.12 ^a^	5.90 ± 0.97 ^bc^	12.47 ± 0.12 ^c^	2.29 ± 0.05 ^c^
Pterostilbene	9.85 ± 0.26 ^b^	5.09 ± 0.86 ^bc^	16.46 ± 0.42 ^b^	2.70 ± 0.18 ^ab^

^1^ Water fraction from the ethanol extract of the embryo of *Nelumbo nucifera* Gaertn. (NEW), stilbene glycoside from *Pleuropterus multiflorus* (Thunb.) Turcz. ex Nakai. (PM-SG). ^2^ DPPH IC_50_: concentration of antioxidant required to scavenge 50% of DPPH radicals. ^3^ TEAC IC_50_: concentration of sample (expressed as mM Trolox equivalents) required to achieve 50% inhibition of ABTS•^+^ radical cation absorbance. ^4^ ORAC IC_50_: concentration of sample (expressed as mM Trolox equivalents) required to inhibit 50% of the peroxyl radical-induced fluorescence decay, calculated from the net area under the fluorescence decay curve (AUC) after subtracting blank AUC. ^5^ Reducing power IC_50_: concentration of sample required to produce an absorbance of 0.5 at 700 nm in the ferric reducing assay; lower values indicate stronger reducing capacity. Each value represents mean ± SD from three different experiments (each experiment was conducted in triplicate). Data in the same column with different letters are significantly different at *p* < 0.05.

**Table 2:** Cytotoxicity on HepG2 of different samples.

Sample	Concentration	Toxicity
	(μg/mL)	Cell Viability (%)
Control	—	100.0 ± 3.4
Control (1% DMSO)	—	98.2 ± 1.0 ^a^
NEW	31.3	94.3 ± 4.4 ^ab^
	62.5	97.6 ± 1.2 ^a^
	125	97.4 ± 0.9 ^a^
	250	82.0 ± 3.2 ^c^
	500	72.1 ± 1.3 ^d^
PM-SG	31.3	98.7 ± 3.9 ^ab^
	62.5	97.8 ± 1.0 ^a^
	125	97.6 ± 1.0 ^a^
	250	96.1 ± 1.8 ^a^
	500	77.0 ± 2.6 ^c^
Pterostilbene	3.13	96.9 ± 0.7 ^ab^
	6.31	98.0 ± 0.5 ^a^
	12.5	99.0 ± 0.8 ^a^
	25.0	9.52 ± 4.5 ^e^
	50.0	0.64 ± 0.5 ^f^

Each value represents mean ± SD from three independent experiments (each performed in triplicate). Statistical significance was determined by one-way ANOVA followed by Tukey’s post-hoc test at *p* < 0.05. Within each column, values with different superscript letters (a, b, c, ...) are significantly different (*p* < 0.05). NEW was compared with the untreated control group, while PM-SG and pterostilbene were compared with the 1% DMSO control group.

**Table 3:** IC_50_ values for the inhibition of peroxyl radical-induced DCFH-DA oxidation by quercetin, EGCG, and different samples in the cellular model.

Sample ^1^	IC_50_ (μg/mL)		CV (%)
Quercetin	3.21 ± 0.2 ^ab^	≈10.62 ± 0.88 (μM)	8.29
EGCG	2.74 ± 0.18 ^ab^	≈5.39 ± 0.38 (μM)	7.13
NEW	457.83 ± 34.33 ^d^	—	7.50
PM-SG	211.35 ± 34.11 ^c^	—	16.14
Pterostilbene	>12.5	<48.8 ^2^ (μM)	—

^1^ Water fraction from the ethanol extract of the embryo of *Nelumbo nucifera* Gaertn. (NEW), stilbene glycoside from *Pleuropterus multiflorus* (Thunb.) Turcz. ex Nakai (PM-SG). Each value represents mean ± SD from two or three different experiments (each experiment was conducted in triplicate). Data with different letters are significantly different at *p* < 0.05 (one-way ANOVA). ^2^ IC_50_ for pterostilbene was not reached within the non-cytotoxic concentration range (≤12.5 µg/mL); the upper-bound value (<48.8 µM) is calculated from the cytotoxicity ceiling (MW = 256.3 g/mol).

**Table 4:** Physicochemical and pharmacokinetic descriptors of principal bioactive compounds.

Compound	Molecular Formula	MW (g/mol)	logP	HBA	HBD	TPSA (Ų)	Rotatable Bonds	Oral Bioavailability (%) ^1,^*	Cell Permeability (% in 2h) ^2^	SIRT1 (IC_50_) ^3^
Neferine	C_38_H_44_N_2_O_6_	624.8	4.12	8	2	75.6	6	6.4 ± 1.2	8.3 ± 2.1	>50
TSG	C_20_H_22_O_9_	406.4	1.23	9	6	150.1	5	18.2 ± 3.4	16.1 ± 3.8	12.5 ± 2.3
Pterostilbene	C_16_H_16_O_3_	256.3	3.48	3	1	41.5	3	80–95	92.3 ± 4.2	3.8 ± 0.7
Multiflorumiside H	C_40_H_44_O_18_	812.25	1.98	14	10	198.2	8	~8–10	~9.4 ± 2.8	>30
Resveratrol ^4^	C_14_H_12_O_3_	228.2	3.14	3	3	60.7	2	20–30	35.4 ± 6.1	7.2 ± 1.5

^1^ References [7,25,27,29]. ^2^ Cellular uptake measured in Caco-2 or HepG2 cells. ^3^ References [28,29]. ^4^ Resveratrol included as a reference compound for structure-activity comparison. * Bioavailability and cell permeability estimated from TPSA-based prediction model; SIRT1 IC_50_ estimated from structural analogy with TSG dimers; values are approximations and not directly measured in this study.

## Data Availability

The original contributions presented in this study are included in the article/Supplementary Materials. Further inquiries can be directed to the corresponding author.

## Supplementary Materials

The following supporting information can be downloaded at: https://www.mdpi.com/article/10.3390/molecules31172981/s1. Table S1: LC-MS spectrometric data of multiflorumisides H–L in PM-SG extracts. Table S2: Phenolic compound quantification in N-E-W and PM-SG extracts. Table S3: QSAR model statistics for cellular antioxidant activity. Table S4: Predicted versus experimental cellular antioxidant activity (CAA) values. Table S5: Input parameters for the lifespan extension QSAR model. Table S6: QSAR model statistics for lifespan extension. Table S7: Predicted versus experimental lifespan extension values. Figure S1: LC-MS/MS chromatographic analysis of multiflorumisides in PM-SG extract. Figure S2: Chemical structures of the main compounds identified in NEW and PM-SG extracts.

## Abbreviations

AAI: Anti-Aging index; AUC: Area Under the Curve; CAA: Cellular Antioxidant Activity; CT: Cycle Time; DCFH DA: 2′,7′ dichlorofluorescin diacetate; DPPH: 2,2-Diphenyl-1-picrylhydrazyl; EC: epicatechin; EGCG: Epigallocatechin-3-gallate; EUK8: A Synthetic Salen-Manganese Complex; HBA: Number of Hydrogen Bond Acceptors; HBD: Number of Hydrogen Bond Donors; HepG2: Human Hepatocellular Carcinoma Cell Line; HSF-1: Heat Shock Factor 1; HSP: Heat Shock Protein; HSP-16.2: Heat Shock Protein 16.2 kDa; HSP-70: Heat Shock Protein 70 kDa; HSP-90: Heat Shock Protein 90 kDa; IC_50_: Half-Maximal Inhibitory Concentration; LC-MS: Liquid Chromatography–Tandem Mass Spectrometry; logP: Logarithm of octanol-water partition coefficient; *m*/*z*: mass-to-charge ratio; mTOR: Mammalian Target of Rapamycin; MW: Molecular Weight; NAD+: Nicotinamide Adenine Dinucleotide (oxidized form); NADPH: Nicotinamide Adenine Dinucleotide Phosphate (reduced form); NEW: *Nelumbo nucifera Gaertn*. embryo (seed plumule) extract–water-soluble fraction; NF-κB: Nuclear Factor Kappa B; NGM: Nematode Growth Medium; Nrf2: Nuclear Factor Erythroid 2-Related Factor 2; ORAC: Oxygen Radical Absorbance Capacity; PBS: Phosphate Buffered Saline; PCA: Principal Component Analysis; PM-SG: *Pleuropterus multiflorus* (Thunb.) Turcz. ex Nakai (syn. *Polygonum multiflorum* Thunb.) stilbene glycoside; Q^2^: Cross-Validated Correlation Coefficient; RMSE: Root Mean Square Error; ROS: Reactive Oxygen Species; HPLC: High-Performance Liquid Chromatography; SAR: Structure–Activity Relationship; SASP: Senescence-Associated Secretory Phenotype; SGLT1: Sodium-Glucose Linked Transporter 1; SIR-2.1: Sirtuin-2.1 (*C. elegans* ortholog of mammalian SIRT1); SIRT1: Sirtuin 1 (NAD^+^-dependent deacetylase); TE: Trolox Equivalents; TEAC: Trolox Equivalent Antioxidant Capacity; TIC: Total Ion Chromatogram; TPSA: Topological Polar Surface Area; TSG: 2,3,5,4-Tetrahydroxystilbene-2-*O*-β-*D*-glucoside.
