# Supplementary material for: Multi-Level Evaluation of Antioxidant Activity, Aging-Related Physiological Functions, and Longevity Effects of Nelumbo nucifera Gaertn. Embryo Extract, Pleuropterus multiflorus (Thunb.) Turcz. ex Nakai Stilbene Glycoside, and Pterostilbene
Source: Molecules. 2026 Aug 26;31(17):2981. doi: 10.3390/molecules31172981 (PMC13567345; doi:10.3390/molecules31172981)
Supplement: Supplementary file 1 [file molecules-31-02981-s001.zip › molecules-4333075-supplementary.pdf]

Supplementary material

Table S1. LC-MS/MS parameters and major fragment ions of quantified compounds in NEW and PM-SG extracts.

Compound	Molecular Formula	Precursor Ion (m/z)	Major Fragment Ions (m/z)	Proposed Assignment
TSG	$C_{20}H_{22}O_9$	429.1156 $[M+H]^+$ 407.1337 $[M+Na]^+$	243.0656 $[M+H–glu]^+$ 225.0550 $[–H_2O]$ 201.0550 $[C_8H_9O_4]^+$ 159.0446 $[C_{10}H_7O_2]^+$	Aglycone Dehydrated aglycone Resorcinol-type Tropylium-like
Multiflorumiside H	$C_{40}H_{44}O_{18}$	812.2499 $[M+H]^+$ 835.2422 $[M+Na]^+$	651.1712 $[M+H–glu]^+$ 489.1185 $[–2glu]^+$ 243.0656 (TSG aglycone) 225.0550	Dimeric stilbene - glu Core fragment TSG unit Dehydrated
Multiflorumiside I	$C_{40}H_{44}O_{18}$	812.2496 $[M+H]^+$ 835.2419 $[M+Na]^+$	651.1705, 489.1178, 243.0655 (similar to H)	Isomer of H
Multiflorumiside J	$C_{40}H_{44}O_{19}$	828.2472 $[M+H]^+$ 851.2395 $[M+Na]^+$	667.1660, 505.1132, 243.0656	Additional OH group
Multiflorumiside K	$C_{60}H_{66}O_{27}$	1218.3772 $[M+H]^+$ 1241.3695 $[M+Na]^+$	1057.3041 [–glu] 895.2415 [–2glu] 651.1710, 243.0656	Trimeric stilbene
Multiflorumiside L	$C_{60}H_{66}O_{27}$	1218.3740 $[M+H]^+$ 1241.3663 $[M+Na]^+$	1057.3010, 895.2390, 651.1695, 243.0654	Isomer of K

Compound	Molecular Formula	Precursor Ion (m/z)	Major Fragment Ions (m/z)	Proposed Assignment
Neferine	$C_{38}H_{44}N_2O_6$	625.3272 $[M+H]^+$	473.2435 [$-C_9H_{10}O_2$]<br>429.2172 [$-C_{11}H_{14}O_3$]<br>206.1176 (benzylisoquinoline)<br>175.0757 (dopamine)	Loss of dimethoxyphenyl<br>Further cleavage<br>Core fragment<br>Dopamine-related
Liensinine	$C_{37}H_{42}N_2O_6$	611.3121 $[M+H]^+$	459.2278 [$-C_9H_{10}O_2$]<br>415.2015 [$-CO_2$]<br>206.1176, 174.0913	Similar to neferine
Isoliensinine	$C_{37}H_{42}N_2O_6$	611.3121 $[M+H]^+$	459.2278, 415.2015,<br>192.1019 [isoquinoline]<br>160.0757 $[C_{10}H_{10}NO]^+$	Isomer-specific
Kaempferol	$C_{15}H_{10}O_6$	285.0399 $[M-H]^-$	257.0450 [–CO]<br>229.0501 [–2CO]<br>151.0031 [$1,3A^-$]<br>107.0132 [B-ring]	Retro-Diels–Alder
Quercetin	$C_{15}H_{10}O_7$	301.0348 $[M-H]^-$	273.0401 [–CO]<br>245.0451 [–2CO]<br>179.0015 [$1,3A^-$]<br>151.0031 [$1,2A^-$]	RDA fragments
Isorhamnetin	$C_{16}H_{12}O_7$	315.0505 $[M-H]^-$	300.0274 [$-CH_3$]<br>271.0245 [$-CO-CH_3$]<br>151.0031 [A-ring]<br>107.0132 [B-ring]	Methylated quercetin
Pterostilbene	$C_{16}H_{16}O_3$	255.1021 $[M-H]^-$	240.0786 [$-CH_3$]<br>225.0550 [–2CH3]<br>211.0755 [$-C_2H_4$]<br>183.0806 [$-C_3H_4O_2$]	Successive demethylation

All m/z values are calculated monoisotopic masses.

Table S2. Chemical composition of NEW and PM-SG extracts with fragmentation patternscts

	**NEW (μg/g)**	**PM-SG [*] (μg/g)**
Liensinine	2140 ± 85.14[c]	—
Isoliensinine	1487 ± 57.42[de]	—
Neferine	2874 ± 124.1[a]	—
Kaempferol	2437 ± 78.67[b]	—
Quercetin	1420 ± 75.14[de]	—
Isorhamnetin	1230 ± 64.42[f]	—
2,3,5,4'-tetrahydroxystilbene-2-*O*-β-D-glucoside (TSG)	—	14270 ± 175.1[a]
Multiflorumiside H	—	275.8 ± 11.45[b]
Multiflorumiside I	—	134.4 ± 5.712[c]
Multiflorumiside J	—	107.2 ± 3.472[d]
Multiflorumiside K	—	30.72 ± 2.742[e]
Multiflorumiside L	—	20.64 ± 1.247[f]

[*] Purity (85%) refers to the relative peak area of TSG in the HPLC-DAD chromatogram. Concentrations in this table are expressed in μg/g of extract and are not equivalent to peak area percentages; Data are presented as mean ± standard deviation (SD), and concentrations are expressed in μg/g of extract. "—" indicates not detected. Different lowercase letters (a, b,

c, d) within the same row denote significant differences (Tukey's HSD test, $p < 0.05$). For the five multiflorumisides (H–L), which are not commercially available as individual reference standards, semi-quantification was performed using the external calibration curve of TSG as a surrogate. This approach is justified by their similar molecular weight class, shared stilbenoid core structure, and expected comparable electrospray ionization response. Therefore, all values for multiflorumisides are reported as TSG equivalents (μg/g), in line with published methods for quantifying oligomeric stilbenes in *Polygonum multiflorum* matrices.

Table S3. QSAR model for chemical antioxidant activity

Parameter	Coefficient	Std. Error	t-value	p-value	95% CI
Intercept	2.87	0.42	6.83	0.003	1.89–3.85
logP	0.83	0.18	4.61	0.009	0.42–1.24
$logP^2$	–0.12	0.03	–4.00	0.016	–0.19—0.05
TPSA	–0.024	0.006	–4.00	0.016	–0.037—0.011
HBD	–0.15	0.05	–3.00	0.039	–0.27—0.03

Table S4. QSAR model for cellular antioxidant activity and AAI validation

Compound	Experimental EC_{50} (μg/mL)	Predicted EC_{50} (μg/mL)	Residual	% Error
Neferine	457.83	428.6	+29.2	6.4%
TSG	211.35	223.8	–12.5	5.9%
Pterostilbene	<12.5	11.2	–	–
Quercetin*	3.21	3.8	–0.6	18.7%

*External validation compound

Table S5. AMPK activation scores and QSAR model for lifespan extensio

Compound	Bioavailability (%)	logP	1/SIRT1 EC_{50} (μM^{-1})	AMPK Score	MW/100	Observed Extension (%)	Predicted Extension (%)
Neferine	6.4	4.12	<0.02	0	6.25	18–22 (mid: 20.0)	18.7
TSG	18.2	1.23	0.08	1	4.06	25–35 (mid: 30.0)	31.4
Pterostilbene	87.5	3.48	0.26	3	2.56	40–50 (mid: 45.0)	46.2
Resveratrol*	25.0	3.14	0.14	2	2.28	~32.0 (lit.)	30.8
Multiflorumiside H	~8–10	1.98	<0.03 ‡	1	8.12	15–18 (mid: 16.5)	14.2

*Reference compound from literature [2,7,23,32].; AMPK scores: 0 = no activation; 1 = weak (EC_{50} > 10 μM); 2 = moderate (5–10 μM); 3 = strong (< 5 μM).

Table S6. QSAR model statistics for lifespan extension

Parameter	Coefficient	Std. Error	Standardized β	t-value	p-value
Intercept	5.2	2.1	–	2.48	0.089
Bioavailability	0.42	0.06	0.68	7.00	0.002
logP	12.3	2.4	0.52	5.13	0.007
$logP^2$	–2.1	0.4	–0.48	–5.25	0.006
1/SIRT1 EC_{50}	8.7	1.8	0.41	4.83	0.009
AMPK score	6.4	1.2	0.35	5.33	0.006
MW/100	–3.2	0.9	–0.22	–3.56	0.024

Table S7. Predicted vs. experimental lifespan extension

Compound	Experimental (%)	Predicted (%)	Residual (%)	% Error
Neferine	20.0	18.7	+1.3	6.5%
TSG	30.0	31.4	–1.4	4.7%
Pterostilbene	45.0	46.2	–1.2	2.7%
Resveratrol*	32.0 (lit.)	30.8	+1.2	3.8%
Multiflorumiside H	16.5	14.2	+2.3	6.5%

*Reference compound from literature [2,7,23,32].

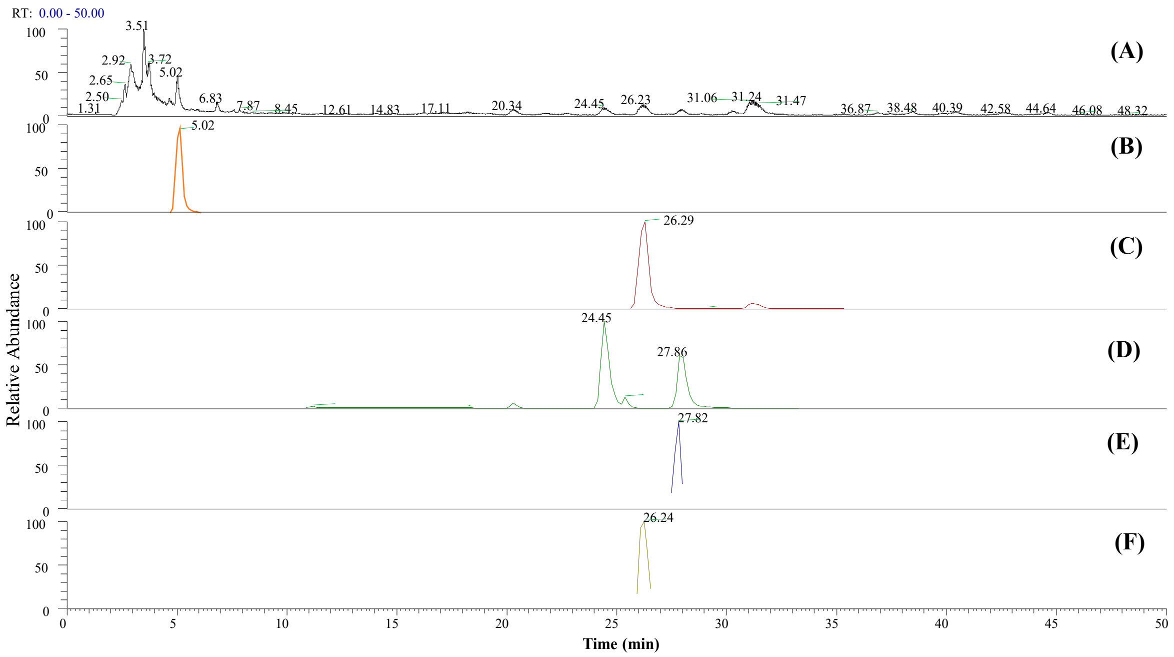

Figure S1. Liquid Chromatography-Tandem Mass Spectrometry (LC-MS/MS) Analysis of multiflorumisides in PM-SG Extract. (A) Total ion chromatogram (TIC), **(B)** SRM for TSG, **(C)** SRM for Multiflorumiside J, **(D)** SRM for Multiflorumiside H and I, **(E)**, SRM for Multiflorumiside L **(F)** SRM for Multiflorumiside K.

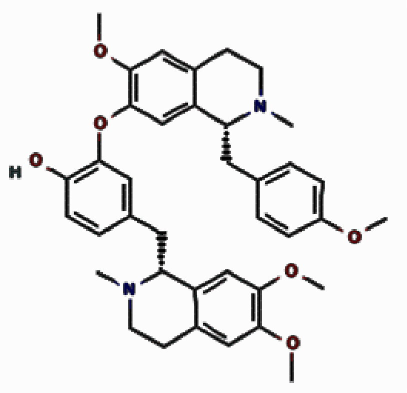

Neferine

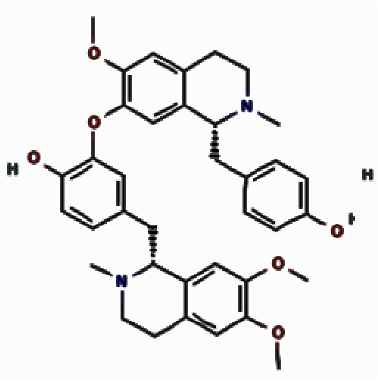

Liensinine

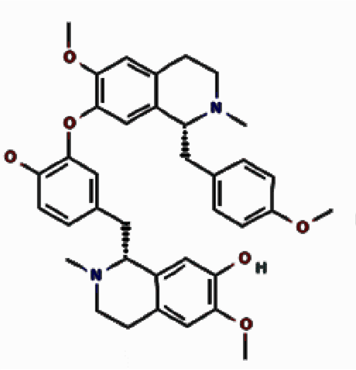

Isoliensinine

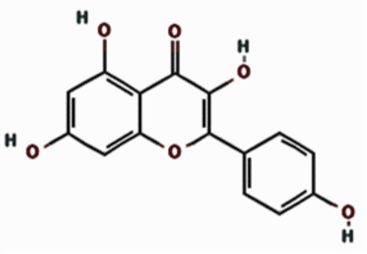

Kaempferol

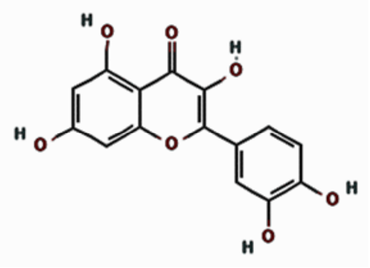

Quercetin

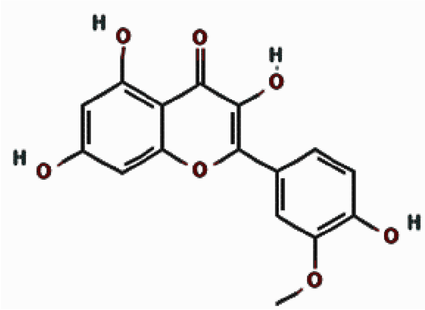

Isorhamnetin

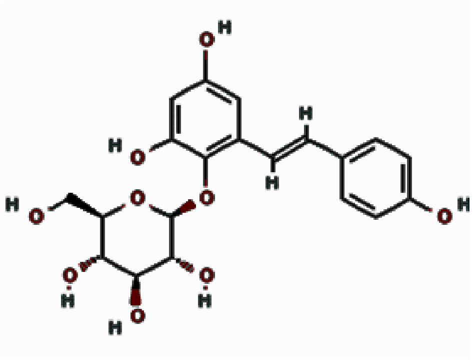

Tetrahydroxystilbene (TSG)

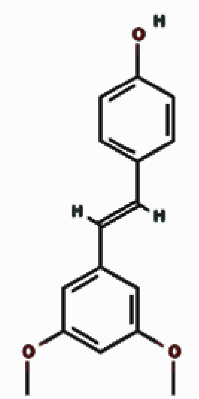

Pterostilbene

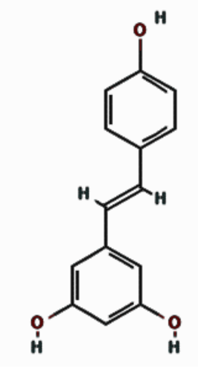

Resveratrol

Figure S2: Chemical structures of the main compounds identified in NEW and PM-SG (neferine, liensinine, isoliensinine, kaempferol, quercetin, isorhamnetin, TSG, pterostilbene, resveratrol, and multiflorumiside H)
